# Genome sequence of the acid-tolerant *Burkholderia sp.* strain WSM2232 from Karijini National Park, Australia

**DOI:** 10.4056/sigs.5018795

**Published:** 2013-11-15

**Authors:** Robert Walker, Elizabeth Watkin, Rui Tian, Lambert Bräu, Graham O’Hara, Lynne Goodwin, James Han, Tatiparthi Reddy, Marcel Huntemann, Amrita Pati, Tanja Woyke, Konstantinos Mavromatis, Victor Markowitz, Natalia Ivanova, Nikos Kyrpides, Wayne Reeve

**Affiliations:** 1 School of Biomedical Sciences, Faculty of Health Sciences, Curtin University, Western Australia, Australia; 2 Centre for Rhizobium Studies, School of Veterinary and Life Sciences, Murdoch University, Western Australia, Australia; 3 School of Life and Environmental Sciences, Deakin University, Victoria, Australia; 4 Los Alamos National Laboratory, Bioscience Division, Los Alamos, New Mexico, USA; 5 DOE Joint Genome Institute, Walnut Creek, California, USA; 6 Biological Data Management and Technology Center, Lawrence Berkeley National Laboratory, Berkeley, California, USA

**Keywords:** root-nodule bacteria, nitrogen fixation, rhizobia, *Betaproteobacteria*

## Abstract

*Burkholderia sp.* strain WSM2232 is an aerobic, motile, Gram-negative, non-spore-forming acid-tolerant rod that was trapped in 2001 from acidic soil collected from Karijini National Park (Australia) using *Gastrolobium capitatum* as a host. WSM2232 was effective in nitrogen fixation with *G. capitatu*m but subsequently lost symbiotic competence during long-term storage. Here we describe the features of *Burkholderia sp.* strain WSM2232, together with genome sequence information and its annotation. The 7,208,311 bp standard-draft genome is arranged into 72 scaffolds of 72 contigs containing 6,322 protein-coding genes and 61 RNA-only encoding genes. The loss of symbiotic capability can now be attributed to the loss of nodulation and nitrogen fixation genes from the genome. This rhizobial genome is one of 100 sequenced as part of the DOE Joint Genome Institute 2010 Genomic Encyclopedia for *Bacteria* and *Archaea*-Root Nodule Bacteria (GEBA-RNB) project.

## Introduction

*Burkholderia* spp. are a diverse group of organisms capable of thriving in diverse environments with many forming mutualistic associations with organisms such as fungi and plants [1]. The development in the 1960s and 1970s of a rational classification system for *Pseudomonas* species resulted in proposals to give different generic names to taxonomically distinct groups. The organisms previously classified within *Pseudomonas* rRNA similarity Group II were transferred into the new genus *Burkholderia* [2]. All described *Burkholderia* species at that time were phytopathogenic, or opportunistic mammalian pathogens with the type species *B. cepacia* becoming a growing community health concern in immunocompromised and cystic fibrosis patients [3-5]. With the isolation of more *Burkholderia* spp., it has become apparent that the genus is a far more complex mix, with the isolation of numerous soil-inhabiting species capable of degrading heavy metals and environmental contaminants [6,7]. Further reports identified plant growth promoting (PGP) species and legume microsymbionts. This led to a paradigm shift in rhizobiology and resulted in numerous new novel *Burkholderia* spp. descriptions [8-10].

Most PGP, or legume microsymbiont species of *Burkholderia* have been isolated in South America from Mimosa spp. or South Africa from *Papilionoideae* legumes and until recently, *B. graminis* was the only described PGP bacterial species isolated from Australia in the maize rhizosphere [11]. Australian *Burkholderia* have been isolated as nodule occupants from some Acacia spp., [12] however none have been authenticated or tested for the nodulation of other legumes. There is little data regarding the symbiosis between *Burkholderia* and legumes in Australia compared to South Africa and South America. *Burkholderia sp.* WSM2232 was trapped from acidic soil (pHCaCl2 4.8) collected from Karijini National Park (Western Australia) using *Gastrolobium capitatum* as a host. Sites where the soil pH was higher (pH_CaCl2_ >7) did not contain any *Burkholderia* symbionts but did contain numerous *Bradyrhizobium* and *Rhizobium* spp. (Watkin, unpublished). Soil pH is an edaphic variable that controls microbial biogeography [13] and the acid tolerance of *Burkholderia* has been shown to account for the biogeographical distribution of this genus [14].

The symbiotic capacity of WSM2232 was authenticated in axenic glasshouse trials using inoculation of *G. capitatum* grown in nitrogen free conditions. Inoculated plants nodulated by WSM2232 produced significantly greater mass than uninoculated controls. WSM2232 was subcultured and placed in long-term storage in frozen laboratory glycerol stocks. Isolate revival and inoculation onto endemic Australian legumes failed to elicit a symbiotic response. The reason for the loss of the symbiotic phenotype has, until now, not been identified.

The genome of *Burkholderia* strain WSM2232 is one of two Australian *Burkholderia* genomes (the other being that of WSM2230 (GOLD ID Gi08831)) that have now been sequenced through the Genomic Encyclopedia for *Bacteria* and *Archaea*-Root Nodule Bacteria (GEBA-RNB) program. Here we present a preliminary description of the general features of *Burkholderia sp.* WSM2232 together with its genome sequence and annotation. The absence of nodulation genes within this genome explains the nodulation minus symbiotic phenotype of the laboratory cultured strain. The genomes of WSM2232 and WSM2230 will be an important resource to identify the processes enabling such isolates to adapt to the infertile, highly acidic soils that dominate the Australian landscape.

## Classification and features

**Figure 1:**
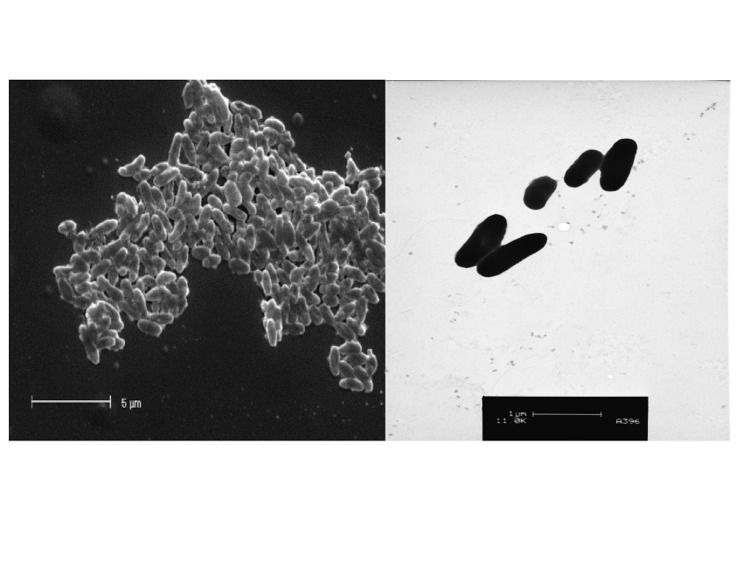
Images of *Burkholderia sp.* strain WSM2232using scanning (A) and transmission (B) electron microscopy.

*Burkholderia sp.* strain WSM2232 is a motile, non-sporulating, non-encapsulated, Gram-negative rod in the order *Burkholderiales* of the class *Betaproteobacteria*. The rod-shaped form varies in size with dimensions of 0.25-0.5 μm for width and 0.5-2.0 μm for length (Figure 1A and 1B).

It is fast growing, forming colonies within 1-2 days when grown on LB agar [15] devoid of NaCl and within 3-4 days when grown on half strength Lupin Agar (½LA) [16], tryptone-yeast extract agar (TY) [17] or a modified yeast-mannitol agar (YMA) [18] at 28°C. Colonies on ½LA are opaque, slightly domed and moderately mucoid with smooth margins.

*Burkholderia sp.* WSM2232 falls into a large clade containing PGP, bioremediation and legume microsymbiont species, and WSM2232 demonstrates PGP phenotypes including phosphate solubilization and hydroxamate-like siderophore production and is acid tolerant with growth in the pH range of 4.5-9.0 (Walker, unpublished).

**Figure 2:**
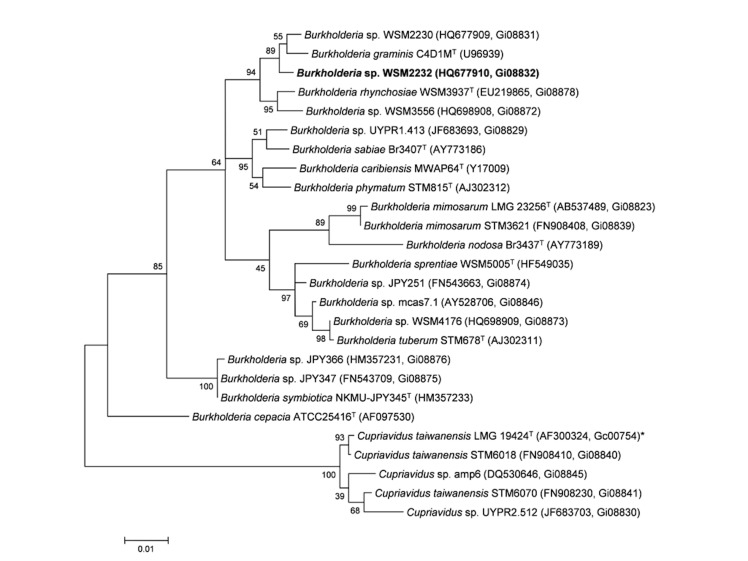
Phylogenetic tree showing the relationship of *Burkholderia sp.* strain WSM2232 (shown in bold print) to other members of the order *Burkholderiales* based on aligned sequences of the 16S rRNA gene (1,242 bp internal region). All sites were informative and there were no gap-containing sites. Phylogenetic analyses were performed using MEGA [29], version 5. The tree was built using the Maximum-Likelihood method with the General Time Reversible model [30]. Bootstrap analysis [31] with 500 replicates was performed to assess the support for the clusters. Type strains are indicated with a superscript T. Brackets after the strain name contain a DNA database accession number and/or a GOLD ID (beginning with the prefix G) for a sequencing project registered in GOLD [32]. Published genomes are indicated with an asterisk.

Minimum Information about the Genome Sequence (MIGS) is provided in Table 1. Figure 2 shows the phylogenetic neighborhood of *Burkholderia sp.* strain WSM2232 in a 16S rRNA sequence based tree. This strain shares 99% (1352/1364 bp) sequence identity to the 16S rRNA gene of the sequenced strain *Burkholderia sp.* WSM2230 (Gi08831).

**Table 1:** Classification and general features of *Burkholderia sp.* strain WSM2232 according to the MIGS recommendations [19]

**MIGS ID**	**Property**	**Term**	**Evidence code**
	Current classification	Domain *Bacteria*	TAS [20]
		Phylum *Proteobacteria*	TAS [21]
		Class *Betaproteobacteria*	TAS [22,23]
		Order *Burkholderiales*	TAS [23,24]
		Family *Burkholderiaceae*	TAS [23,25]
		Genus *Burkholderia*	TAS [2,26,27]
		Species *Burkholderia sp.*	IDA
		Strain WSM2232	IDA
	Gram stain	Negative	IDA
	Cell shape	Rod	IDA
	Motility	Motile	IDA
	Sporulation	Non-sporulating	NAS
	Temperature range	Mesophile	IDA
	Optimum temperature	30°C	IDA
	Salinity	Non-halophile	IDA
MIGS-22	Oxygen requirement	Aerobic	IDA
	Carbon source	Varied	IDA
	Energy source	Chemoorganotroph	NAS
MIGS-6	Habitat	Soil, root nodule, on host	IDA
MIGS-15	Biotic relationship	Free living, symbiotic	IDA
MIGS-14	Pathogenicity	Non-pathogenic	IDA
	Biosafety level	1	TAS
	Isolation	Root nodule of *Gastrolobium capitatum*	IDA
MIGS-4	Geographic location	Karijini National Park, Australia	IDA
MIGS-5	Soil collection date	September, 2001	IDA
MIGS-4.1 MIGS-4.2	Latitude Longitude	117.99 -22.45	IDA IDA
MIGS-4.3	Depth	0-10 cm	IDA
MIGS-4.4	Altitude	Not recorded	IDA

Evidence codes – IDA: Inferred from Direct Assay; TAS: Traceable Author Statement (i.e., a direct report exists in the literature); NAS: Non-traceable Author Statement (i.e., not directly observed for the living, isolated sample, but based on a generally accepted property for the species, or anecdotal evidence). These evidence codes are from the Gene Ontology project [28].

### Symbiotaxonomy

*Burkholderia sp.* WSM2232 formed nodules (Nod+) and fixed N_2_ (Fix+) with *G. capitatum* when first isolated and was Nod- on various other Australian legumes and *Mimosa pudica* (Table 2). However, after long-term storage and subsequent culture, it failed to effectively nodulate *G. capitatum*.

**Table 2:** Compatibility of *Burkholderia sp.* WSM2232 with nine legume species for nodulation (Nod) and N_2_-Fixation (Fix).

**Species Name**	**Common Name**	**Growth Type**	**Nod**	**Fix**	**Reference**
*Gastrolobium capitatum*	Bitter Pea	Perennial	+^1^	+^1^	IDA
			-^2^	-^2^	IDA
*Kennedia coccinea*	Coral Vine	Perennial	-	-	IDA
*Swainsona formosa*	Sturts Desert Pea	Annual	-	-	IDA
*Indigofera trita*	-	Annual	-	-	IDA
*Oxylobium robustum*	Shaggy Pea	Perennial	-	-	IDA
*Acacia acuminata*	Jam Wattle	Perennial	-	-	IDA
*Acacia paraneura*	Weeping Mulga	Perennial	-	-	IDA
*Acacia stenophylla*	-	Perennial	-	-	IDA
*Mimosa pudica*	Sensitive Plant	Perennial	-	-	IDA

^1^ result obtained from trapping experiment

^2^ authentication result following long-term storage

IDA: Inferred from Direct Assay from http://www.geneontology.org/GO.evidence.shtml of the Gene Ontology project [28].

### Phenotype Microarray

Strain WSM2232 was assayed using the Biolog Phenotype Microarray® plates (PM1 to 3) system testing 190 carbon and 95 nitrogen compounds. Plates were purchased from Biolog and tests were carried out per manufacturer’s instructions. The irreversible reduction of tetrazolium dye to formazan is used in this system to report on active metabolism [33]. The results obtained from the colorimetric assay are shown in Table 3.

**Table 3:** Reduction of tetrazolium dye by NADH produced by respiring cells of *Burkholderia sp.* WSM2232 in the Biolog Phenotype Microarray

**PM1 plate** **Compound**		**PM2 plate** **Compound**		**PM3 plate** **Compound**	
L-Arabinose	+	Chondroitin Sulfate C	-	Ammonia	+
N-Acetyl-D Glucosamine	+	α-Cyclodextrin	-	Nitrite	+
D-Saccharic Acid	+	β-Cyclodextrin	-	Nitrate	+
Succinic Acid	+	γ-Cyclodextrin	-	Urea	+
D-Galactose	+	Dextrin	+	Biuret	-
L-Aspartic Acid	+	Gelatin	-	L-Alanine	+
L-Proline	+	Glycogen	-	L-Arginine	+
D-Alanine	+	Inulin	-	L-Asparagine	+
D-Trehalose	+	Laminarin	-	L-Aspartic Acid	+
D-Mannose	+	Mannan	-	L-Cysteine	+
Dulcitol	+	Pectin	-	L-Glutamic Acid	+
D-Serine	-	N-Acetyl-D-Galactosamine	+	L-Glutamine	+
D-Sorbitol	+	N-Acetyl-Neuraminic Acid	-	Glycine	+
Glycerol	+	β-D-Allose	-	L-Histidine	+
L-Fucose	+	Amygdalin	-	L-Isoleucine	+
D-Glucuronic Acid	+	D-Arabinose	+	L-Leucine	+
D-Gluconic Acid	+	D-Arabitol	+	L-Lysine	+
D,L-α-Glycerol-Phosphate	+	L-Arabitol	+	L-Methionine	+
D-Xylose	+	Arbutin	-	L-Phenylalanine	+
L-Lactic Acid	+	2-Deoxy-D-Ribose	+	L-Proline	+
Formic Acid	+	I-Erythritol	-	L-Serine	+
D-Mannitol	+	D-Fucose	+	L-Threonine	+
L-Glutamic Acid	+	3-0-β-D-Galacto-pyranosyl-DArabinose	-	L-Tryptophan	+
D-Glucose-6-Phosphate	+	Gentiobiose	-	L-Tyrosine	+
D-Galactonic Acid-γ-Lactone	+	L-Glucose	-	L-Valine	+
D,L-Malic Acid	+	Lactitol	-	D-Alanine	+
D-Ribose	+	D-Melezitose	-	D-Asparagine	+
Tween 20	+	Maltitol	-	D-Aspartic Acid	+
L-Rhamnose	+	α-Methyl-D-Glucoside	-	D-Glutamic Acid	+
D-Fructose	+	β-Methyl-D-Galactoside	+	D-Lysine	+
Acetic Acid	+	3-Methyl Glucose	-	D-Serine	+
α-D-Glucose	+	β-Methyl-D-Glucuronic Acid	-	D-Valine	+
Maltose	-	α-Methyl-D-Mannoside	-	L-Citrulline	+
D-Melibiose	-	β-Methyl-D-Xyloside	-	L-Homoserine	+
Thymidine	-	Palatinose	-	L-Ornithine	+
L-Asparagine	+	D-Raffinose	-	N-Acetyl-D,L-Glutamic Acid	+
D-Aspartic Acid	-	Salicin	-	N-Phthaloyl-L-Glutamic Acid	-
D-Glucosaminic Acid	+	Sedoheptulosan	-	L-Pyroglutamic Acid	+
1,2-Propanediol	-	L-Sorbose	-	Hydroxylamine	+
Tween 40	+	Stachyose	-	Methylamine	+
α-Keto-Glutaric Acid	+	D-Tagatose	+	N-Amylamine	+
α-Keto-Butyric Acid	+	Turanose	+	N-Butylamine	+
α-Methyl-D-Galactoside	-	Xylitol	+	Ethylamine	-
α-D-Lactose	-	N-Acetyl-D-Glucosaminitol	+	Ethanolamine	+
Lactulose	+	γ-Amino Butyric Acid	+	Ethylenediamine	-
Sucrose	-	δ-Amino Valeric Acid	+	Putrescine	+
Uridine	+	Butyric Acid	+	Agmatine	-
L-Glutamine	+	Capric Acid	-	Histamine	-
M-Tartaric Acid	+	Caproic Acid	+	β-Phenylethylamine	+
D-Glucose-1-Phosphate	+	Citraconic Acid	+	Tyramine	-
D-Fructose-6-Phosphate	+	Citramalic Acid	+	Acetamide	+
Tween 80	+	D-Glucosamine	+	Formamide	+
α-Hydroxy Glutaric Acid-γ-Lactone	-	2-Hydroxy Benzoic Acid	-	Glucuronamide	+
α-Hydroxy Butyric Acid	+	4-Hydroxy Benzoic Acid	+	D,L-Lactamide	+
β-Methyl-D-Glucoside	-	β-Hydroxy Butyric Acid	+	D-Glucosamine	+
Adonitol	+	γ-Hydroxy Butyric Acid	+	DGalactosamine	+
Maltotriose	-	α-Keto Valeric Acid	-	DMannosamine	+
2-Deoxy Adenosine	-	Itaconic Acid	-	N-Acetyl-D-Glucosamine	+
Adenosine	+	5-Keto-D-Gluconic Acid	-	N-Acetyl-D-Galactosamine	-
Glycy-L-Aspartic Acid	+	D-Lactic Acid Methyl Ester	+	N-Acetyl-D-Mannosamine	-
Citric Acid	+	Malonic Acid	+	Adenine	+
M-Inositol	+	Melibionic Acid	+	Adenosine	+
D-Threonine	-	Oxalic Acid	+	Cytidine	+
Fumaric Acid	+	Oxalomalic Acid	+	Cytosine	+
Bromo Succinic Acid	+	Quinic Acid	+	Guanine	-
Propionic Acid	+	D-Ribono-1,4-Lactone	-	Guanosine	+
Mucic Acid	+	Sebacic Acid	+	Thymine	+
Glycolic Acid	-	Sorbic Acid	+	Thymidine	-
Glyoxylic Acid	+	Succinamic Acid	+	Uracil	+
D-Cellobiose	-	D-Tartaric Acid	+	Uridine	+
Inosine	+	L-Tartari c Acid	+	Inosine	+
Glycyl-L-Glutamic Acid	+	Acetamide	-	Xanthine	+
Tricarballylic Acid	+	L-Alaninamide	+	Xanthosine	+
L-Serine	+	N-Acetyl-L-Glutamic Acid	+	Uric Acid	+
L-Threonine	+	L-Arginine	+	Alloxan	+
L-Alanine	+	Glycine	-	Allantoin	+
L-Allnyl-Glycine	+	L-Histidine	+	Parabanic Acid	+
Acetoacetic Acid	+	L-Homoserine	+	D,L-α-Amino-N-Butyric Acid	+
N-Acetyl-β-D-Mannosamine	-	Hydroxy-L-Proline	+	γ-Amino-N-Butyric Acid	+
Mono Methyl Succinate	+	L-Isoleucine	+	ε-Amino-N-Caproic Acid	-
Methyl Pyruvate	+	L-Leucine	+	D,L-α-Amino-Caprylic Acid	-
D-Malic Acid	+	L-Lysine	+	δ-Amino-N-Valeric Acid	+
L-Malic Acid	+	L-Methionine	-	α-Amino-N-Valeric Acid	+
Glycyl-L-Proline	+	L-Ornithine	+	Ala-Asp	+
p-Hydroxy Phenyl Acetic Acid	+	L-Phenylalanine	+	Ala-Gln	+
m-Hydroxy Phenyl Acetic Acid	-	L-Pyroglutamic Acid	+	Ala-Glu	+
Tyramine	-	L-Valine	+	Ala-Gly	+
D-Psicose	-	D,L-Carnitine	+	Ala-His	+
L-Lyxose	+	Sec-Butylamine	-	Ala-Leu	+
Glucuronamide	-	D,L-Octopamine	-	Ala-Thr	+
Pyruvic Acid	+	Putrescine	-	Gly-Asn	+
L-Galactonic Acid-γ-Lactone	+	Dihydroxy Acetone	-	Gly-Gln	+
D-Galacturonic Acid	+	2,3-Butanediol	+	Gly-Glu	+
Phenylethylamine	+	2,3-Butanone	+	Gly-Met	+
2-Aminoethanol	+	3-Hydrox y-2-Butanone	-	Met-Ala	+

## Genome sequencing and annotation information

### Genome project history

This organism was selected for sequencing on the basis of its environmental and agricultural relevance to issues in global carbon cycling, alternative energy production, and biogeochemical importance, and is part of the Community Sequencing Program at the U.S. Department of Energy, Joint Genome Institute (JGI) for projects of relevance to agency missions. The genome project is deposited in the Genomes OnLine Database [32] and a standard-draft genome sequence in IMG. Sequencing, finishing and annotation were performed by the JGI. A summary of the project information is shown in Table 4.

**Table 4:** Genome sequencing project information for *Burkholderia sp.* WSM2232.

**MIGS ID**	**Property**	**Term**
MIGS-31	Finishing quality	Standard draft
MIGS-28	Libraries used	One Illumina fragment library
MIGS-29	Sequencing platforms	Illumina HiSeq 2000
MIGS-31.2	Sequencing coverage	Illumina: 255×
MIGS-30	Assemblers	Velvet version 1.1.04; Allpaths-LG version r37348
MIGS-32	Gene calling methods	Prodigal 1.4
	GOLD ID	Gi08832^a^
	NCBI project ID	182741
	Database: IMG	2508501125^b^
	Project relevance	Symbiotic N_2_ fixation, agriculture

### Growth conditions and DNA isolation

*Burkholderia sp.* strain WSM2232 was cultured to mid logarithmic phase in 60 ml of TY rich medium on a gyratory shaker at 28°C [34]. DNA was isolated from the cells using a CTAB (Cetyl trimethyl ammonium bromide) bacterial genomic DNA isolation method (http://my.jgi.doe.gov/general/index.html).

### Genome sequencing and assembly

The genome of *Burkholderia sp.* strain WSM2232 was sequenced at the Joint Genome Institute (JGI) using Illumina technology [35]. An Illumina standard shotgun library was constructed and sequenced using the Illumina HiSeq 2000 platform, which generated 12,244,888, reads totaling 1,837 Mbp.

All general aspects of library construction and sequencing performed at the JGI can be found at http://my.jgi.doe.gov/general/index.html. All raw Illumina sequence data was passed through DUK, a filtering program developed at JGI, which removes known Illumina sequencing and library preparation artifacts (Mingkun, L., Copeland, A. and Han, J., unpublished). The following steps were then performed for assembly:

1. Filtered Illumina reads were assembled using Velvet [36] (version 1.1.04)
2. 1–3 Kbp simulated paired end reads were created from Velvet contigs using wgsim (https://github.com/lh3/wgsim)
3. Illumina reads were assembled with simulated read pairs using Allpaths–LG [37] (version r37348).

Parameters for assembly steps were:

1. Velvet --v --s 51 --e 71 --i 2 --t 1 --f "-shortPaired -fastq $FASTQ" --o "-ins_length 250 -min_contig_lgth 500")
2. wgsim (-e 0 -1 76 -2 76 -r 0 -R 0 -X 0)
3. Allpaths–LG (STD_1,project,assembly,fragment,1,200,35,,,inward,0,0 SIMREADS,project,assembly,jumping,1,,,3000,300,inward,0,0).

The final draft assembly contained 72 contigs in 72 scaffolds. The total size of the genome is 7.2 Mbp and the final assembly is based on 1,837 Mbp of Illumina data, which provides an average 255× coverage of the genome.

### Genome annotation

Genes were identified using Prodigal [38] as part of the DOE-JGI annotation pipeline [39], followed by a round of manual curation using the JGI GenePrimp pipeline [40]. The predicted CDSs were translated and used to search the National Center for Biotechnology Information (NCBI) nonredundant database, UniProt, TIGRFam, Pfam, PRIAM, KEGG, COG, and InterPro databases. The tRNAScanSE tool [41] was used to find tRNA genes, whereas ribosomal RNA genes were found by searches against models of the ribosomal RNA genes built from SILVA [42]. Other non–coding RNAs such as the RNA components of the protein secretion complex and the RNase P were identified by searching the genome for the corresponding Rfam profiles using INFERNAL (http://infernal.janelia.org). Additional gene prediction analysis and manual functional annotation was performed within the Integrated Microbial Genomes (IMG-ER) platform (http://img.jgi.doe.gov/er) [43].

## Genome properties

**Figure 3:**
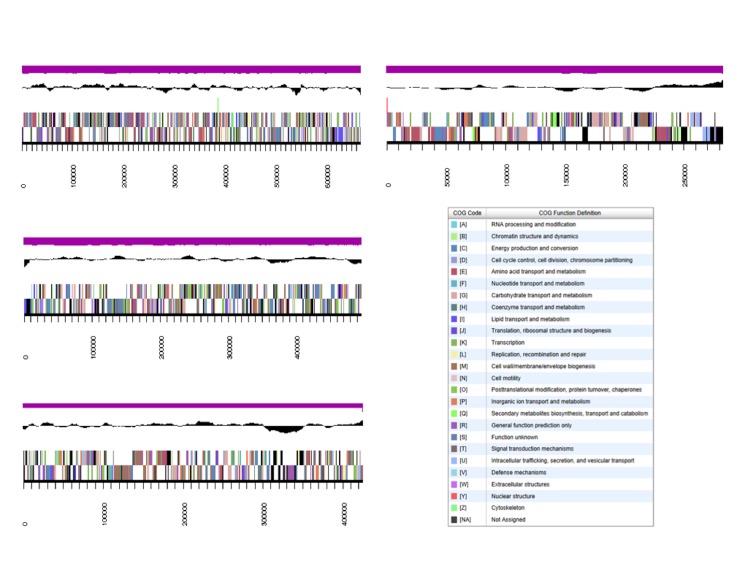
Graphical map of the four largest scaffolds genome for the genome of *Burkholderia sp.* strain WSM2232. From bottom to the top of each scaffold: Genes on forward strand (color by COG categories as denoted by the IMG platform), Genes on reverse strand (color by COG categories), RNA genes (tRNAs green, sRNAs red, other RNAs black), GC content, GC skew.

The genome is 7,208,311 nucleotides 63.11% GC content (Table 5) and comprised of 72 scaffolds (Figure 3) of 72 contigs. From a total of 6,383 genes, 6,322 were protein encoding and 61 RNA only encoding genes. The majority of genes (80.90%) were assigned a putative function whilst the remaining genes were annotated as hypothetical. The distribution of genes into COGs functional categories is presented in Table 6.

**Table 5:** Genome Statistics for *Burkholderia sp.* strain WSM2232

**Attribute**		**Value**		**% of Total**	
Genome size (bp)	7,208,311		100.00	
DNA coding region (bp)	6,203,174		86.06	
DNA G+C content (bp)	4,548,885		63.11	
Number of scaffolds	72			
Number of contigs	72			
Total gene	6,383		100.00	
RNA genes	61		0.96	
rRNA operons*	1		0.02	
Protein-coding genes	6,322		99.04	
Genes with function prediction	5,164		80.90	
Genes assigned to COGs	5,151		80.70	
Genes assigned Pfam domains	5,425		84.99	
Genes with signal peptides	645		10.10	
Genes with transmembrane helices	1,497		23.45	
CRISPR repeats	1			

*4 copies of 5S, 2 copies of 16S and 1 copy of 23S rRNA.

**Table 6:** Number of protein coding genes of *Burkholderia sp.* strain WSM2232 associated with the general COG functional categories.

**Code**	**Value**	**%age**	**Description**
J	474	8.15	Carbohydrate transport and metabolism
A	3	0.05	RNA processing and modification
K	151	2.60	Replication, recombination and repair
L	559	9.61	Transcription
B	1	0.0	Chromatin structure and dynamics
D	42	0.72	Cell cycle control, cell division and chromosome partioning
Y	0	0.0	Nuclear structure
V	0	0.0	Defense mechcanism
T	318	5.47	Signal transduction mechanisms
M	371	6.38	Cell wall/membrane/envelope biogenesis
N	125	2.15	Cell motility
Z	0	0.00	Cytoskeleton
W	2	0.03	Extracellular structures
U	154	2.65	Intracellular trafficking, secretion, and vesicular transport
O	183	3.15	Posttranslational modification, protein turnover, chaperones
C	384	6.60	Energy production conversion
G	194	3.34	Translation, ribosomal structure and biogenesis
E	569	9.79	Amino acid transport and metabolism
F	100	1.72	Nucleotide transport and metabolism
H	213	3.66	Coenzyme transport and metabolism
I	277	4.76	Lipid transport and metabolism
P	269	4.63	Inorganic ion transport and metabolism
Q	199	3.42	Secondary metabolite biosynthesis, transport and catabolism
R	673	11.58	General function prediction only
S	500	8.60	Function unknown
-	1,232	19.30	Not in COGs

Acknowledgements
